# Impact of Heating Exposure on the Micro-Push-Out Bond Strength of Bioceramic Sealers

**DOI:** 10.1155/2023/3327275

**Published:** 2023-10-31

**Authors:** Guilherme Rodrigues Cândido Lopes, Maria Eduarda Paz Dotto, Lincon Hideo Nomura, Daniela Peressoni Vieira Schuldt, Lucas da Fonseca Roberti Garcia, Cleonice da Silveira Teixeira

**Affiliations:** ^1^Department of Dentistry, Endodontics Division, Health Sciences Center, Federal University of Santa Catarina, Florianopolis, Santa Catarina, Brazil; ^2^Department of Endodontics, College of Dental Medicine, Nova Southeastern University, Clearwater, FL, USA

## Abstract

**Introduction:**

The aim of this study was to evaluate the effect of heating of bioceramic and epoxy resin-based sealers on their micro-push-out bond strength (BS) to root canal dentin.

**Methods:**

After criterial selection, 30 human teeth were decoronated and 1-mm thick slices (*n* = 60) were obtained perpendicularly along tooth axis, from cervical and middle root thirds, with a diamond disc attached to a cutting machine. In each slice, three 1.0-mm diameter orifices were made. After rinsing with 17% EDTA and 2.5% NaOCl, each orifice was filled with Bio-C Sealer or BioRoot RCS or AH Plus, according to the manufacturers' instructions. After filling, half of the slices (*n* = 30) were heated at 100°C for 1 min, and the other half were kept at room temperature. After 7 days-controlled storage, micro-push-out test was performed in a Universal Testing Machine. Failures were analyzed using a stereomicroscope. Statistical analysis was performed with One-Way ANOVA and post hoc Tukey (*α* = 5%) tests.

**Results:**

AH Plus demonstrated higher BS values after heating (*p*=0.001) when compared to nonheated. The other sealers did not show a statistically significant difference (*p* > 0.05). When heated, the average BS values for AH Plus were higher than for BioRoot RCS and Bio-C Sealer (*p* < 0.001). Cohesive failure mode was the most frequent, followed by adhesive and mixed ones.

**Conclusion:**

Heating provided a higher push-out BS to root dentin for AH Plus and did not influence BioRoot RCS or Bio-C sealer.

## 1. Introduction

Filling procedures in endodontics are performed with gutta-percha and a sealer [1]. The sealer is necessary to fill the empty spaces between gutta-percha and the root canal walls [1]. Epoxy resin-based sealers are widely used in endodontic practice due to their favorable physical–chemical properties [2]. AH Plus (Dentsply Sirona, York, PA, USA) stands as the primary representative of this category of sealers [3]. It is an epoxy resin-based sealer that is known to have proper dimensional stability, flowability, high-bond strength (BS) to root canal dentin, biocompatibility, and radiopacity [3].

Recently, another category of sealer has gained significant attention—the bioceramics or calcium silicate-based sealers [4]. These sealers result from the combination of calcium silicate, calcium phosphate, amorphous silicon dioxide, and tantalum pentoxide [4]. Bioactivity is the main characteristic of these sealers, which is the material ability to induce mineralized tissue formation [5], favoring periapical tissue repair [6]. BioRoot RCS (Septodont, Saint-Maur-des Fosses, France) is a powder/liquid hydraulic tricalcium silicate-based cement recommended for single-cone or cold lateral condensation techniques [7]. The powder contains tricalcium silicate, povidone, and zirconium oxide, and the liquid is a calcium chloride and polycarboxylate aqueous solution. Studies reported that BioRoot RCS is bioactive [8], has lower cytotoxicity than conventional root canal sealers [9] and has antimicrobial activity [10]. Recently, a new calcium silicate-based sealer, the Bio-C Sealer (Angelus, Londrina, Brazil), was commercially launched in the market. According to the manufacturer, it is an injectable, ready-to-use sealer containing calcium silicates, calcium aluminate, calcium oxide, zirconium oxide, iron oxide, silicon dioxide, and dispersing agent in its composition. When it was compared to TotalFill and AH Plus, Bio-C sealer showed a shorter setting time, alkalinization ability, adequate flow, and radiopacity, as well as low-volumetric change [11]. Moreover, Bio-C Sealer and TotalFill BC Sealer demonstrated better cytocompatibility in terms of cell viability, migration, morphology, attachment, and mineralization capacity, than AH Plus [12].

In a traditional obturation technique, known as “continuous waves of condensation [13],” heat is generated to plasticize the gutta-percha and increase its ability to adapt to the complex root canal anatomy, as well as to improve the quality and homogeneity of the obturation [14]. In this technique, an electronic equipment generates and transmits heat from a metallic tip to the gutta-percha and the sealer [14]. A concern is that the heat can alter the physicochemical properties of endodontic sealers [1, 14]. A study demonstrated that when an epoxy resin-based sealer was heated at higher temperatures, and/or with a long exposure time, it was associated with cement degradation [15]. When calcium silicate-based sealers were heated to a 100°C temperature, it significantly changed its flowability, setting time and solubility [16]. However, the effective BS and adhesive interface created between the filling material and the root canal dentin, considering these changes in temperature during filling procedures have not been properly studied.

Considering the above, the purpose of this study was to assess the effect of heating exposure on the BS of bioceramic and epoxy resin-based sealers to root dentin. The null hypothesis posits that heating exposure does not have impact on the BS of the sealers to the root dentin.

## 2. Material and Methods

### 2.1. Sample Size and Selection

The present research was previously approved by the Human Research Ethics Committee (protocol number 3.621.326). Single-rooted human teeth, extracted by unrelated reasons from this study. Sample size was based on the previous studies with similar objectives and calculated using the G  ^*∗*^ Power 3.1.9.6 software for Windows (Universitat Kiel, Kiel, Germany) with a *α* = 0.05 and 90% statistical power. A total number of 26 specimens was calculated and rounded up to 30 specimens.

After teeth visual and radiographical evaluation, the following inclusion criteria were adopted: complete apical root formation, no root curvatures above 5°, no decays, resorptions, calcifications, cracks, or fractures. After cleaning and disinfection, the teeth were kept in 0.9% saline solution and 4°C temperature.

### 2.2. Sample Preparation and Micro-Push-Out Test

**Figure 1:**
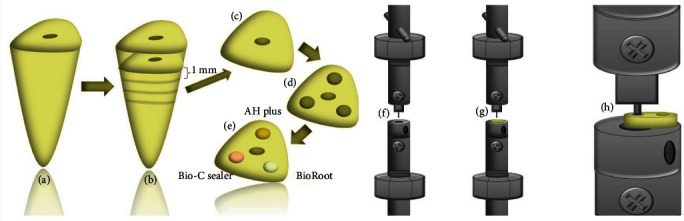
Schematic representation of the specimens' preparation for the micro-push-out test. Specimen (root) after crown removal (a). Cutting of the cervical and middle root thirds into dentin slices (b). 1-mm slices (c). Slice after performing the micro-push-out cavities (d). Cavities filled with AH Plus (yellow), BioRoot RCS (white) e o Bio-C Sealer (pink) (e). Schematic representation of push-out bond strength test (f–h).

The teeth were individually fixed in acrylic plates to allow the cut for slices in a Universal Machine Test (Isomet 1000, Buehler, Lake Forest, USA).  Figure 1(a)–1(d) presents the schematic representation of the specimens' preparation for the micro-push-out test. About 1-mm thickness slices from cervical and middle root thirds were obtained, perpendicular to each tooth long axis (Figures 1(a) and 1(b)). Each slice was measured with a pachymeter (Vernier, China) and three cavities were made with a 1.2-mm diameter cylindrical diamond bur (#3145, Microdont, San Bernardino, California, USA) attached in a high-speed pen and under water/air spray. Orifices were made parallel to the root canal walls, keeping a 1-mm distance between each space (Figures 1(c) and 1(d)). The samples were rinsed with 17% ethylenediaminetetraacetic acid (EDTA) (Merck, Darmstadt, Germany) for 1 min, 2,5% sodium hypochlorite for 1 min, and dried with paper points (Cell Pack, Dentsply, York, PA, USA).

The slices were individually fixed with wax in a glass plate and randomly distributed into two groups (*n* = 30): heated (G_heat_) and nonheated (G_room_). Sealers were mixed according to the manufacturer's instructions and the cavities were filled with AH Plus, BioRoot RCS, and Bio-C sealer, until a slight excess was observed. Shortly after filling the cavities, the specimen in G_heat_ were heated to a temperature of 100°C for 1 min in an oven. In G_room_ they were kept at room temperature (25°C). Then, a polyester strip and a glass plate were placed on top of the specimens, held by a clamp, to remove sealer excess and flatten the surfaces. Finally, the samples were kept in an oven at 37°C and 100% relative humidity for 7 days. After this period, samples were demounted from the glass plates and the surfaces were flattened with a 600-grit sandpaper under running water.

One slice at a time was individually attached to a stainless-steel base plate containing a 2.0-mm diameter hole at its center, fixed to the lower portion of the Universal Testing Machine (Instron model 4444, Instron Corp, Canton, USA). A metallic cylindrical plunger with a cross-section of 0.8 mm in diameter, was positioned centrally over the sealer in each cavity, with the load activated in the apical–coronal direction. The test was performed with a crosshead speed of 0.5 mm per min until bond failure occurred (Figure 1(f)–1(h)). The maximum load at the displacement moment was measured in kilo-Newtons (kN), processed in Newtons (N), and converted into MPa (Mega Pascal), by dividing the force (F) by the cavity lateral area (SL). The sealer adhesion area to root dentin was calculated using the formula: SL = 2*πr* × *h*, where *π* = constant 3.14, *r* = cavity radius, and *h* = material height.

### 2.3. Failure Mode Analysis

The slices were examined under a stereomicroscope, with magnifications up to ×100 (SteREO Discovery.V12, Carl Zeiss, Jena, Germany). Failure mode was classified into three subtypes: adhesive failure-dentin surface free of sealer; cohesive failure-material fracture, and dentin walls still covered by sealer; and mixed failure-part of the dentin walls remained covered by sealer.

### 2.4. Statistical Analysis

Data normality was verified by the Kolmogorov–Smirnov test and the homoscedasticity by the Levene's test. Means association were tested by the Student's *t* test. For analysis of variance, the One-way ANOVA and Tukey post hoc test were performed. Significance level was set at 5%. Statistical software was IBM SPSS 21 Statistic (IBM Corp Armonk, USA).

## 3. Results

**Figure 2:**
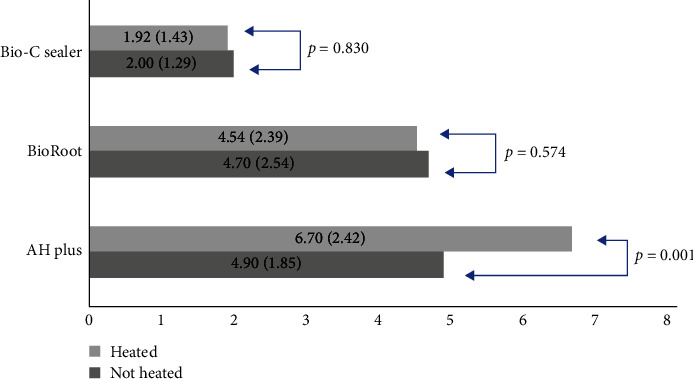
Means (+SD) individual associations between AH Plus, BioRoot and Bio-C Sealer heated and non-heated (Student's *t* test) (*p* < 0.05).

Means and standard deviations (SD) of shear BS by micro-push-out of AH Plus, BioRoot and Bio-C Sealer cements are shown in Figure 2. There was a statistically significant difference when comparing heated and nonheated AH Plus groups (*p* < 0.05).

**Figure 3:**
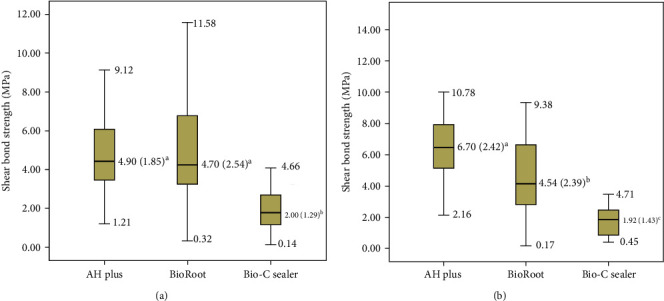
Means, SD, maximal, and minimal confidence interval values for nonheated sealers micro-push-out bond strength box plot in MPa. Different lowercase letters indicate significant difference between each group (*n* = 30) (a). Means, SD, maximal, and minimal confidence interval values for heated sealers micro-push-out bond strength box plot in MPa. Different lowercase letters indicate significant difference between each group (*n* = 30) (b).

Post hoc analysis demonstrated statistically significant difference between the unheated groups of AH Plus and Bio-C Sealer (*p* < 0.001) and BioRoot RCS and Bio-C Sealer (*p* < 0.001) (Figure 3(a)). When the sealers were heated, statistically significant differences were observed between AH Plus and BioRoot RCS (*p*=0.001), AH Plus and Bio-C Sealer (*p* < 0.001) and BioRoot RCS and Bio-C Sealer (*p* < 0.001) (Figure 3(b)).  Table 1 shows the failures modes according to groups (G_heat_ or G_room_) and sealer used. The most frequent failure type was cohesive, followed by adhesive. Mixed failure mode was the least frequent observed.

## 4. Discussion

The results of the present study lead to the rejection of the null hypothesis, as the heating influenced the epoxy resin-based sealer performance, with higher BS to dentin values observed after heating. However, it did not affect the performance of the calcium silicate-based sealers, with similar BS values in both situations (heated and nonheated).

In our study, a temperature of 100°C was used for heating the sealers for 1 min. Thermocompaction devices for gutta-percha, such as the System B, claim to reach temperatures of 200°C. However, it is known that the temperature displayed on the device's screen is not the temperature reached at its applicator tip [17]. Viapiana et al. [17] observed a mean temperature increase (*Δ*°C) at the applicator tip of the System B device, of a maximum of 77.3°C after 1 min from the start of heating. Considering the initial temperature of the tip (temperature of the experimental conditions controlled at 28 ± 0.9°C) in the study, the maximum temperature recorded at the tip of the System B continuous wave plugger was approximately 105.3°C. Therefore, in the present study, a temperature of 100°C was used for the tests in an attempt to approach clinical reality [1]. During the heated vertical compaction, heat is applied in the continuous waves, and the total procedure lasts approximately 1 min, which is why this time was chosen for the application of the mentioned temperature [1].

Push-out BS test is normally performed to assess the shear BS to dentin of root canal fillings or restorative materials [18]. In the conventional push-out test, right after coronal access, root canal preparation, and filling, the root is sectioned perpendicularly along the tooth long axis and the test is performed [2]. However, all stages of endodontic treatment (preparation technique, instruments used, irrigating solutions, materials, and filling procedures employed) can vary greatly and hinder the test standardization [19]. Other biological factors related to the internal canal anatomy can also interfere in the results, such as the structural conditions of the tooth, linked to the presence and quantity of tertiary dentin, pulpal calcifications, canal volume, tooth age, among others [20].

Considering these aspects, in this study we opted for the modified push-out test, which does not use the original root canal space to perform the analysis, but rather orifices made in the root dentin structure in a standardized way [21]. Moreover, the bias of the shaping and filling techniques is reduced and the biological interferences on the study are minimized [19, 21]. However, this methodology also has limitations. These orifices, when filled only with sealers without gutta-percha, allow failure observation only in the sealer and does not simulate the clinical conditions [22]. On the other hand, a positive point is that only the interaction between sealer and dentin is observed, excluding the gutta-percha interference [23].

AH Plus showed the highest values of BS to dentin, which corroborates with the previously published studies that show its good adhesion performance [21, 24, 25]. The AH Plus adhesive capacity has been explained by the covalent bonds between the epoxy resin ring and the amine group, to the existing dentin collagen network [26]. This chemical adhesion, associated with adequate dimensional stability and reduced polymerization stress, results in a stronger bond, and explains the higher BS values of this sealer to dentin, compared to the calcium silicate-based cements [27]. Another point that had influenced this results was the final irrigation, which was carried out with 17% EDTA. This chelator promotes the smear layer removal, increases the occurrence of micro retentions, and facilitates dentin walls drying, which may also have facilitated the hydrophobic sealer penetration, such as AH Plus, into these micro retentions and in dentinal tubules [28].

Interestingly, the epoxy-resin based sealer had greater adhesive resistance after heating, compared to itself when nonheated. This result may have been influenced by the greater flow after heating and a possible imbrication of this cement in root dentin [1, 29]. Studies in the literature have shown that other physical–chemical properties can be affected when this type of cement is heated [30]. According to Qu et al. [30] the setting time of AH Plus was reduced when this material was subjected to a temperature of 140°C. However, another study showed a decrease in this sealer flow after heating, which goes against the assumption that an increase in flow would lead to greater sealer imbrication in the dentinal walls and, consequently, also promoting greater BS values [31]. However, these are two different methodologies. For instance, those authors heated AH Plus cement to a temperature of 97°C, and for 180 s [31].

The bioceramic sealer, BioRoot RCS, showed lower BS values than those obtained by the epoxy resin-based sealer, AH Plus, which corroborates with the results found in the other studies [32, 33]. A possible explanation is that the final irrigation may have played a role on it. A 17% EDTA specimens rinse may have influenced the performance of BioRoot RCS [33]. As already discussed, this chelator decreases the canal walls wetting capacity, which negatively affects the hydrophilic sealers performance, such as the calcium silicate-based ones, messing with the BS between the BioRoot RCS and root dentin [33]. However, the alkalinity of bioceramic cements can compensate for their lower adhesion to root canal walls, while promoting antibacterial activity when in contact with dentin structure, inhibiting proliferation of microorganism in an eventual microleakage [8, 34].

Bio-C Sealer is composed by calcium silicates, calcium aluminate, calcium oxide, zirconium oxide, iron oxide, silicon dioxide, and dispersing agent [34]. It is presented as premixed paste and disposed in an individual ready for use syringe [12]. According to the manufacturer, this material has a high pH, a setting time of 4 hr, is biocompatible and has adequate radiopacity [34]. In the present study, the specimens filled with this sealer had the lowest BS values to dentin, regardless the occurrence of heating. Another study that evaluated Bio-C Sealer at 100°C, found a significant reduction in its flow, probably due to dehydration and polyethylene glycol degradation [16]. However, there are few studies available in the literature on this specific sealer, which hinders comparisons to better results understanding [34].

The failure analysis showed a predominance of cohesive failures, regardless of the sealer and group evaluated. Another study, using the conventional push-out test, showed a predominance of cohesive failures when using AH Plus, and mixed failures with BioRoot RCS [24]. The existence of few studies using these bioceramic cements also limits these differences analysis.

One of the main advantages of bioceramic sealers over epoxy resin-based sealers is these materials biocompatibility [34]. However, there is a need for continued research to assess these sealers performance under the different techniques and circumstances, root canal conditions, and clinical situations, to provide greater reliability before recommending these materials use.

## 5. Conclusion

Within the limitations of this *in vitro* study, heating exposure of epoxy resin-based sealer increased its BS to the root dentin. Bioceramic sealers did not have their adhesiveness affected by heating. Most of the bonding failures were the cohesive-type ones, regardless of the heating exposure, as well as the sealer tested.

## Figures and Tables

**Table 1:** Failure mode incidence after the micro-push-out bond strength test (heated and nonheated sealers) (*n* = 30).

Groups	Sealers	Failures		
		Cohesive	Adhesive	Mixed
Nonheated sealers (G_room_)	AH plus	93.33%	3.33%	3.33%
	BioRoot	86.66%	10%	3.33%
	Bio-C sealer	86.66%	6.66%	6.66%

Heated sealers (G_heat_)	AH plus	83.33%	3.33%	13.33%
	BioRoot	76.66%	20%	3.33%
	Bio-C sealer	80%	10%	10%

## Data Availability

The corresponding author can provide access to the datasets generated and/or analyzed during the current study upon a reasonable request.
